# Investigation of pre-structured GaAs surfaces for subsequent site-selective InAs quantum dot growth

**DOI:** 10.1186/1556-276X-6-211

**Published:** 2011-03-11

**Authors:** Mathieu Helfrich, Roland Gröger, Alexander Förste, Dimitri Litvinov, Dagmar Gerthsen, Thomas Schimmel, Daniel M Schaadt

**Affiliations:** 1 DFG-Center for Functional Nanostructures (CFN) and Institut für Angewandte Physik, Karlsruhe Institute of Technology (KIT), 76131 Karlsruhe, Germany; 2 Institute of Nanotechnology (INT) and Institut für Angewandte Physik, Karlsruhe Institute of Technology (KIT), 76131 Karlsruhe, Germany; 3 Laboratorium für Elektronenmikroskopie (LEM), Karlsruhe Institute of Technology (KIT), 76131 Karlsruhe, Germany

## Abstract

In this study, we investigated pre-structured (100) GaAs sample surfaces with respect to subsequent site-selective quantum dot growth. Defects occurring in the GaAs buffer layer grown after pre-structuring are attributed to insufficient cleaning of the samples prior to regrowth. Successive cleaning steps were analyzed and optimized. A UV-ozone cleaning is performed at the end of sample preparation in order to get rid of remaining organic contamination.

## Introduction

Quantum dots (QDs) are promising candidates for quantum information devices such as quantum bits in quantum computers or quantum memories. Self-assembled QDs were investigated in this context during the past decade. They can produce single photons and can be coupled to microcavity resonators [1,2]. However, for large-scale applications it is essential to transfer the aforementioned schemes to well-positioned QDs in order to obtain a defined device architecture. One approach to site-selective QD growth utilizes substrate pre-structuring [3,4]. Small holes are created on the substrate surface in order to alter the surface chemical potential which leads to an increased growth rate at the hole sites. Thus, QDs preferentially nucleate at the defined locations.

Various tools such as electron beam lithography (EBL) or local oxidation are available to pre-structure substrates [5,6]. In most cases the procedure of pre-structuring involves several process steps including different chemicals which influence the substrate surface. For subsequent QD growth, however, it is necessary to provide a clean surface in order to minimize defects and uncontrolled QD nucleation. It is assumed that defects originating from the regrowth interface degrade the optical quality of the QDs. Therefore, great care has to be taken for surface cleaning after pre-structuring.

In this study we investigate the origin and effect of possible surface contamination which occurs during surface pre-structuring.

## Experiment

The samples were grown by molecular beam epitaxy (MBE) and pre-structured using conventional EBL. A GaAs epitaxial layer is grown on epi-ready (100) GaAs wafers followed by surface pre-structuring. During EBL 50-70 nm large holes were defined in a poly(methyl methacrylate)/(methacrylic acid) co-polymer resist on the surface. The holes are arranged on a square grid. Several arrays with varying lattice constants were defined that way. After development the holes were etched down 30 nm by wet chemical etching (WCE) using H_2_SO_4_:H_2_O_2_:H_2_O with a low etch rate of 1 nm/s. The resist was removed and the samples were cleaned in a series of solvent baths and ultrasonic cleaning. An additional cleaning step was introduced later on, which uses ozone generated by ultraviolet light to remove residual organic contamination.

Before QD growth the samples were heated up to 130°C for 1 h in the load lock chamber of the MBE system in order to get rid of volatile surface contamination. The surface oxide was removed *in situ *by Ga-assisted deoxidation [7]. A 16 nm GaAs buffer layer (BL) was then grown at 500°C followed by 1.7 ML of InAs. The growth rates for GaAs and InAs were determined as 0.3 and 0.07 ML/s, respectively.

The pre-structured samples as well as the uncapped QD samples were characterized by atomic force microscopy (AFM). Transmission electron microscopy (TEM) was used in order to investigate the regrowth interface of QDs capped with 80 nm of GaAs.

## Results and discussion

### Sample growth

**Figure 1:**
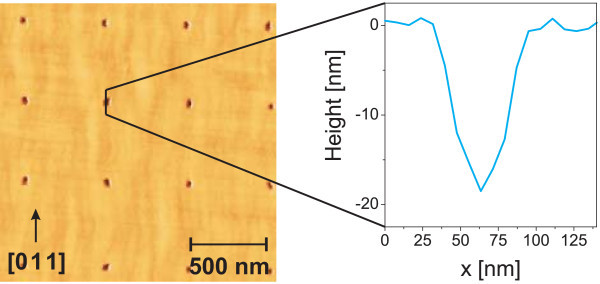
**AFM image of pre-structured GaAs (100) surface**.

Holes with diameters ranging from 50-70 nm are reproducibly defined by EBL and WCE as described above. Figure 1 depicts a pre-structured GaAs sample. The holes are arranged on a square grid with a separation of 500 nm. The representative linescan does not reveal the full depth since the AFM tip is too large to completely enter the hole. Previous calibration of the etch rate suggests a hole depth of about 30 nm for this particular sample.

**Figure 2:**
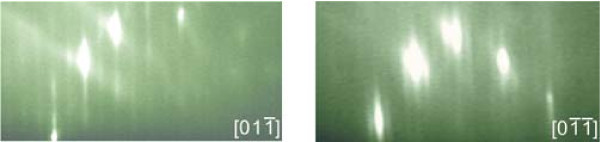
**RHEED pattern of GaAs (100) surface after Ga-assisted deoxidation and subsequent quick anneal under As_4 _atmosphere**. The usual 2 × 4 reconstruction is observed indicating the successful removal of the native oxide.

**Figure 3:**
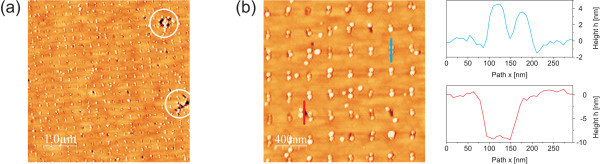
**AFM images of site-selective InAs QDs grown on a pre-structured substrate**. Besides good site-selectivity larger areas of defects are apparent (white circles), **(a)**. Magnified image with linescans of a double dot (top) and a defect hole (bottom), **(b)**.

After reintroducing the pre-structured sample into the MBE chamber, the native oxide has to be removed prior to regrowth. The Ga-assisted deoxidation is advantageous as it is performed at moderate temperatures and thus inhibits additional surface pitting. Since only Ga is provided this gentle deoxidation method does not introduce electronic defects at the surface. Also, any excess Ga on the surface will be incorporated in the subsequent GaAs BL. The surface is monitored by means of *in situ *reflection high energy electron diffraction (RHEED). Diffuse and faint main streaks of the 2 × 4 reconstruction evolve into a clear full 2 × 4 reconstruction pattern after deposition of about 8 ML of Ga. The RHEED pattern of a pre-structured sample after oxide removal is shown in Figure 2. InAs QDs are grown on top of a 16 nm GaAs BL. Mainly double dot nucleation is observed. One possible reason for that phenomenon is a change in hole shape during BL growth with the hole developing into two separate holes with increasing BL thickness [8]. A sample with site-selective InAs QDs is shown in Figure 3. The upper linescan of Figure 3b clearly reveals the double dot feature. Moreover, some defects are apparent in the AFM images as well. Figure 3a shows larger areas of defects (white circles) and Figure 3b contains smaller defect holes, as visualized by the lower linescan. Their origin is further investigated in the following section.

### Hole defects

**Figure 4:**
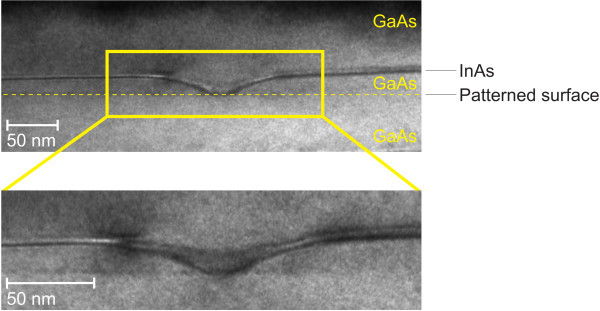
**TEM image of burried defect hole originating from the regrowth interface**.

The additional defect holes were not defined during EBL and thus interfere with the attempt of deterministic QD positioning. The holes are less than 16 nm deep, which corresponds to the BL thickness. That is suggested by the linescan of Figure 3b. Therefore, the defect holes seem to originate from the regrowth interface. Further confirmation is given by TEM analysis of a capped sample. Figure 4 shows a TEM image of the profile of a defect hole, which was found on a pre-structured sample. The different layers of the structure are visible. In this case the defect hole develops from the pre-structured surface upward in the GaAs BL. A local change on that surface inhibits the proper regrowth of GaAs. InAs, however, then nucleates inside the hole, which is finally covered by the final capping layer. The GaAs sidewalls of the defect hole exhibit a curved shape with increasing thickness of the GaAs BL at larger distances from the hole. This implies that strain is accumulated at the surface of the GaAs BL facing the site where nucleation of GaAs is hindered.

In general, two factors can account for the occurrence of the described defect holes. First, incomplete removal of the native oxide could leave residual oxide compounds on the surface which affect the proper GaAs regrowth. Second, insufficient surface cleaning after the lithography process could cause local organic contamination of the sample which also impacts the GaAs growth.

Incomplete deoxidation is rather unlikely since the defect holes are not randomly distributed. Some local areas are found with a high defect density whereas other areas seem very clean. In addition, by controlling the surface evolution during deoxidation using RHEED, it is made sure that enough Ga is provided to completely remove the native oxide. Furthermore, similar samples prepared by conventional thermal deoxidation as well contained comparable defects. That is why we focused on possibility two by analyzing and optimizing the cleaning procedure.

**Figure 5:**
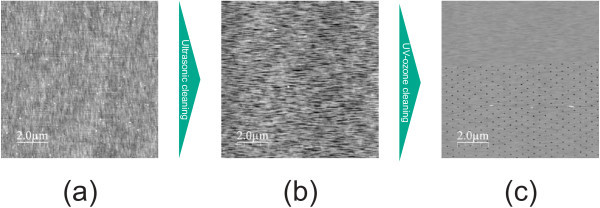
**AFM images of samples at different stages of the cleaning procedure**: after cleaning with solvents **(a)**, using a heated ultrasonic bath **(b)**, and after UV-ozone cleaning **(c)**.

Cleaning samples after EBL comprises several steps. First, the resist needs to be removed which is done with an adequate remover. Thereafter, the sample is cleaned with different solvents (trichlorethylene, acetone, isopropyl alcohol, methanol), if possible in a heated ultrasonic bath. Finally, the samples are rinsed in bi-distilled water. The resist used for EBL contains organic compounds. Especially the high temperature during dry-baking of the resist results in a high stability of such compounds against solvents. Critical steps of the cleaning procedure are depicted in Figure 5. The sample in Figure 5a was cleaned using steps one and two of the above procedure but without ultrasonic bath. A lot of contamination is observed from the AFM image (large particles appearing white). When the samples are cleaned in a heated ultrasonic bath, the amount of contamination is reduced. Especially the amount of smaller particles has decreased, as seen in Figure 5b. However, there are still larger areas of residues remaining on the surface. In order to get rid of these remaining contaminants a UV-ozone cleaning step is introduced. It utilizes a low-pressure mercury lamp that emits radiation at the relevant wavelengths of 184.9 and 253.7 nm [9]. Molecular oxygen is dissociated by the shorter wavelength with the atomic oxygen subsequently forming ozone. Ozone is then decomposed by the longer wavelength. Atomic oxygen is thus constantly provided. In addition, the 253.7 nm radiation excites organic molecules. These react with the atomic oxygen and form simpler, volatile compounds that desorb from the surface. The effect of UV-ozone cleaning is displayed in Figure 5c where essentially all contamination has disappeared. As a result, the number of defect holes should be drastically reduced resulting in a uniform and flat GaAs BL after regrowth. Clean oxygen was fed throughout the cleaning process. UV-ozone cleaning is a very gentle process which does not bombard the surface with ions. The cleaning efficiency is comparable to conventional plasma ashing. However, the costs for appropriate UV-ozone cleaners are much lower. In fact, such devices can easily be self-built.

## Conclusion

In conclusion we have investigated pre-structured GaAs sample surfaces for subsequent site-selective InAs QD growth. We have demonstrated the effect of different cleaning steps after EBL and introduced a UV-ozone cleaning procedure to remove the remaining organic contamination prior to regrowth. Successful operation of this method has been confirmed.

## Abbreviations

AFM: atomic force microscopy; BL: buffer layer; EBL: electron beam lithography; MBE: molecular beam epitaxy; QDs: quantum dots; RHEED: reflection high energy electron diffraction; TEM: transmission electron microscopy; WCE: wet chemical etching.

## Competing interests

The authors declare that they have no competing interests.

## Authors' contributions

MH prepared most of the samples, carried out the experiments to improve the sample surface quality, performed the AFM measurements and drafted the manuscript. RG and AF gave their support with AFM measurements. DL carried out the TEM analysis. DG, TS and DMS conceived of the study and participated in its design and coordination. All authors read and approved the final manuscript.
